# Supplementary material for: Estimating the effect of health assessments on mortality, physical functioning and health care utilisation for women aged 75 years and older
Source: PLoS One. 2021 Apr 2;16(4):e0249207. doi: 10.1371/journal.pone.0249207 (PMC8018643; doi:10.1371/journal.pone.0249207)
Supplement: S1 Fig — a. Model-based survival curves for women with medium mortality risk. b. Model-based survival curves for women with high mortality risk. (PDF) [file pone.0249207.s001.pdf]

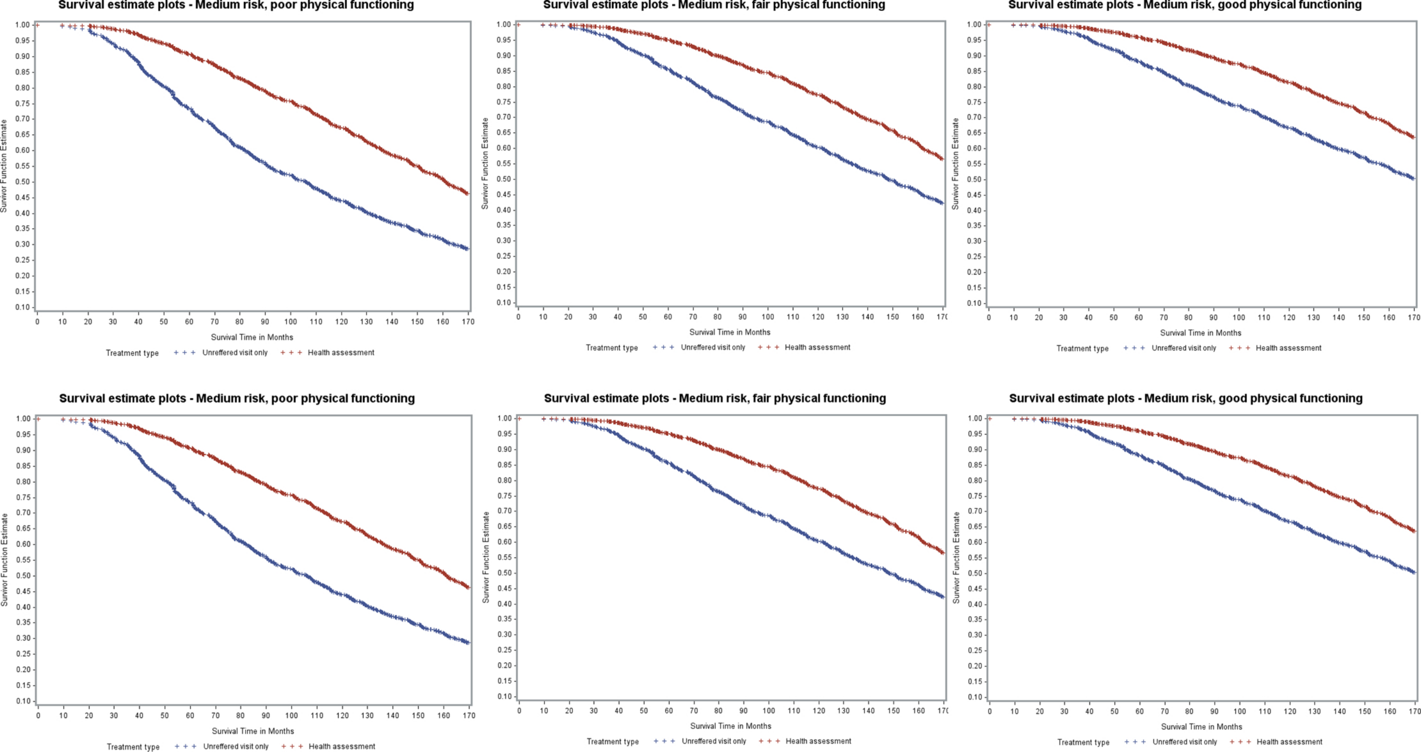
Survival estimate plots - Medium risk, poor physical functioning
Survivor Function Estimate
Survival Time in Months
Treatment type
Unreffered visit only
Health assessment
Survival estimate plots - Medium risk, fair physical functioning
Survivor Function Estimate
Survival Time in Months
Treatment type
Unreffered visit only
Health assessment
Survival estimate plots - Medium risk, good physical functioning
Survivor Function Estimate
Survival Time in Months
Treatment type
Unreffered visit only
Health assessment
Survival estimate plots - Medium risk, poor physical functioning
Survivor Function Estimate
Survival Time in Months
Treatment type
Unreffered visit only
Health assessment
Survival estimate plots - Medium risk, fair physical functioning
Survivor Function Estimate
Survival Time in Months
Treatment type
Unreffered visit only
Health assessment
Survival estimate plots - Medium risk, good physical functioning
Survivor Function Estimate
Survival Time in Months
Treatment type
Unreffered visit only
Health assessment
