# Supplementary figures and images for: Estimating the effect of health assessments on mortality, physical functioning and health care utilisation for women aged 75 years and older
Source: PLoS One. 2021 Apr 2;16(4):e0249207. doi: 10.1371/journal.pone.0249207 (PMC8018643; doi:10.1371/journal.pone.0249207)

## Block 4

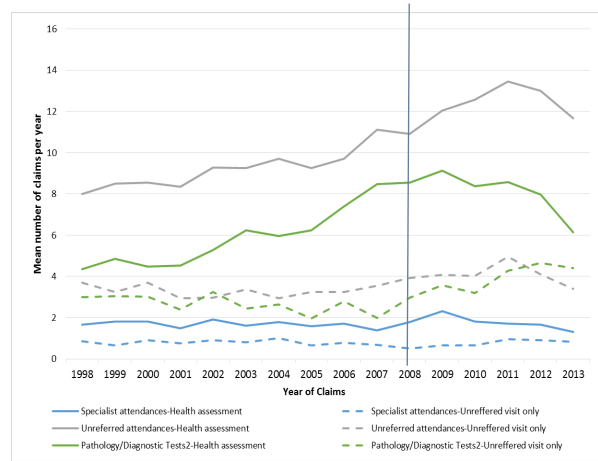

## Block 5

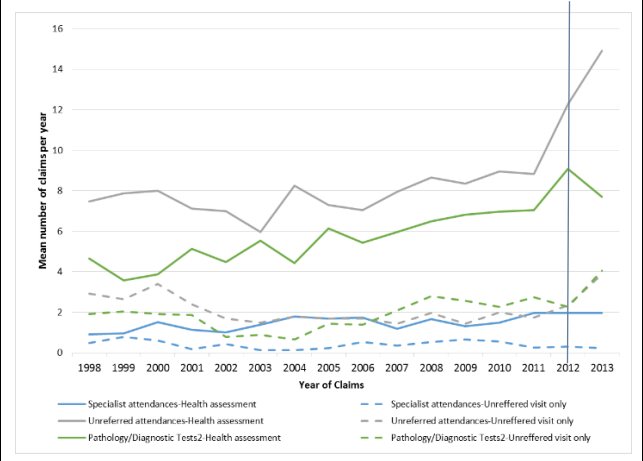

Supplement: S2 Fig — (PDF) [file pone.0249207.s002.pdf]
