# Supplementary material for: Estimating the effect of health assessments on mortality, physical functioning and health care utilisation for women aged 75 years and older
Source: PLoS One. 2021 Apr 2;16(4):e0249207. doi: 10.1371/journal.pone.0249207 (PMC8018643; doi:10.1371/journal.pone.0249207)
Supplement: S1 Table — (PDF) [file pone.0249207.s003.pdf]

Main effect terms	Interaction terms included covariate below with the corresponding main effect term
Age	Age^2
Area of residence	
Smoker	Qualification, heart disease, hypertension, stroke, BMI, marital status
General health	GP, Diabetes, heart disease, asthma, arthritis, hypertension, stroke, BMI, hearing
Qualifications	
Manage on income	
Marital status	Hearing problems, BMI, qualifications, manage on income
Private Health Insurance	Marital status, smoking, qualifications, manage on income
Visits to GP	General health, area of residence, qualification, smoker, manage on income, private health insurance, asthma, arthritis, heart disease, diabetes, stroke, widow, marital status, BMI, urine leakage, physical functioning
Sight problems	
Joints problems	BMI, general health, GP visits, falls
Dizziness	
Hearing problems	Falls, sight problems, general health, age
Falls	BMI, GP visits
Urine leakage	BMI, marital status,
Mental health score	Mental $health^2$
Physical functioning score	Physical $functioning^2$
Doctors diagnosis of:	
Diabetes	BMI,
Heart disease	BMI, hypertension, dizziness
Hypertension	
stroke	
Asthma	BMI,
Arthritis	Joint problems, BMI, falls, smoker, mental health, physical functioning
BMI	
