# Supplementary material for: Estimating the effect of health assessments on mortality, physical functioning and health care utilisation for women aged 75 years and older
Source: PLoS One. 2021 Apr 2;16(4):e0249207. doi: 10.1371/journal.pone.0249207 (PMC8018643; doi:10.1371/journal.pone.0249207)
Supplement: S2 Table — (PDF) [file pone.0249207.s004.pdf]

Characteristic	Low Risk	Medium Risk	High Risk	P-Value
**Treatment type**				
Unreffered visit only	659 (50.0%)	653 (51.1%)	669 (48.9%)	0.541
Health assessment	658 (50.0%)	625 (48.9%)	698 (51.1%)	
**Age distribution**				
70-75 years	999 (75.9%)	285 (22.3%)	31 (2.3%)	<0.001
>75-80 years	310 (23.5%)	943(73.8%)	993 (72.6%)	
>80 years	8 (0.6%)	50 (3.9%)	343 (25.1%)	
**Area of residence**				
Urban	692 (52.5%)	514 (40.2%)	381 (27.9%)	<0.001
Non-Urban	625 (47.5%)	764 (59.8%)	986 (72.1%)	
**GP visits**				
Up to four visits	892 (67.7%)	692 (54.2%)	158 (11.6%)	<0.001
More than four	425 (32.3%)	586 (45.9%)	1209 (88.4%)	
**Morbidities**				
None	687 (52.2%)	322 (25.2%)	133 (9.7%)	<0.001
One	497 (37.7%)	522 (40.9%)	448 (32.8%)	
More than one	133 (10.1%)	434 (33.9%)	786 (57.5%)	
**Joint pain**				
No	657 (49.9%)	707 (55.3%)	767 (56.1%)	0.002
Yes	660 (50.1%)	571 (44.7%)	600 (43.9%)	
