# Supplementary material for: Estimating the effect of health assessments on mortality, physical functioning and health care utilisation for women aged 75 years and older
Source: PLoS One. 2021 Apr 2;16(4):e0249207. doi: 10.1371/journal.pone.0249207 (PMC8018643; doi:10.1371/journal.pone.0249207)
Supplement: Attachment — Submitted filename: Response to Reviewers.pdf [file pone.0249207.s005.pdf]

PRIORITY RESEARCH CENTRE FOR GENERATIONAL HEALTH AND AGEING

THE UNIVERSITY OF NEWCASTLE AUSTRALIA

27 January 2021

Editor in Chief
PLOS One Journal

RE: Rebuttal Letter – Response to Reviewers Comments on Manuscript

To whom it may concern,

Please find enclosed our responses to each point raised by the academic editor and reviewer(s) with regards to our manuscript entitled '*Estimating the effect of health assessments on mortality, physical functioning and health care utilisation for women aged 75 years and older*'.

Comments to the Author

1. Is the manuscript technically sound, and do the data support the conclusions?

The manuscript must describe a technically sound piece of scientific research with data that supports the conclusions. Experiments must have been conducted rigorously, with appropriate controls, replication, and sample sizes. The conclusions must be drawn appropriately based on the data presented.

Reviewer #1: Partly

Reviewer #2: Yes

---

2. Has the statistical analysis been performed appropriately and rigorously?

Reviewer #1: Yes

Reviewer #2: Yes

---

3. Have the authors made all data underlying the findings in their manuscript fully available?

The PLOS Data policy requires authors to make all data underlying the findings described in their manuscript fully available without restriction, with rare exception (please refer to the Data Availability Statement in the manuscript PDF file). The data should be provided as part of the manuscript or its supporting information, or deposited to a public repository. For example, in addition to summary statistics, the data points behind means, medians and variance measures should be available. If there are restrictions on publicly sharing data—e.g. participant privacy or use of data from a third party—those must be specified.

Reviewer #1: Yes

Reviewer #2: No

Please note use of linked administrative and survey data for ALSWH projects is strictly regulated. This strict restriction is due to the inclusion of data from the Australian Government Medicare database, with the ethics and legal approvals requiring that the data can only be accessed by approved researchers and only on site within the ALSWH offices. There are therefore legal and ethical restrictions on sharing the de-identified data set.

De-identified survey data are available to collaborating researchers where a formal request to make use of the material has been approved by the ALSWH Data Access Committee. The committee is receptive of requests for datasets required to replicate results. Information on applying for ALSWH data is available from https://www.alswh.org.au/for-data-users/applying-for-data/.

In order for linked data provided by third parties to be accessed through ALSWH, every data user must be added to the applicable Data Use Agreements and Human Research Ethics Committee protocols.

Data Access enquiries can also be directed to this email: alswh@uq.edu.au.

---

4. Is the manuscript presented in an intelligible fashion and written in standard English?

PLOS ONE does not copyedit accepted manuscripts, so the language in submitted articles must be clear, correct, and unambiguous. Any typographical or grammatical errors should be corrected at revision, so please note any specific errors here.

Reviewer #1: Yes

Reviewer #2: Yes

---

5. Review Comments to the Author

The University of Newcastle
Callaghan NSW 2308 Australia
enquirycentre@newcastle.edu.au
CRICOS Provider Number: 00109J
T +61 2 4921 5000
www.newcastle.edu.au

Please use the space provided to explain your answers to the questions above. You may also include additional comments for the author, including concerns about dual publication, research ethics, or publication ethics. (Please upload your review as an attachment if it exceeds 20,000 characters)

Reviewer #1: #0. What is the strengths and the limitations of this study comparing previous studies to found that health assessment improved the health of older people?
Please clarify it.
(Strengths and limitations in discussion, comparing previous studies)

We have added to the discussion this study goes beyond previous trials to show how assessments are used at a population level and over time, as well as their potential effects on health outcomes. Unlike earlier studies where assessments were conducted within the context of the trial and with controls on the conduct of the assessment and follow-up (eg. Byles et al 2004), this study evaluates assessments according to their uptake by women and to how they are delivered in practice by general practitioners within the health system.

#1. Why did the authors include only women populations?

The study uses data from the Australian Longitudinal Study Women's Health which only includes women. The study was commissioned in response to a recognised need for information on the factors that affect the health of women.

#2. It would be better to summarize what the authors intended to mention in introduction section.

We have summarised the objectives of the analyses in the final paragraph of the introduction.

Reviewer #2: Dear editor,

The manuscript is a longitudinal study that aims to evaluate the impacts of health assessments on long-term care utilisation, physical functioning, and mortality in a cohort of older women aged 75+. The study showed that women who had an assessment had significantly lower odds of poor outcomes than women who had no assessment. However, women who had health assessments also increased healthcare services utilization, which is likely to be an important mechanism in improving the women's health outcomes.

Major Comments

- There are many trials in the literature studying the effects of health assessments on older people's several outcomes. The paper provides a good overview, but it would be interesting to state in the introduction how the paper adds to this branch of the literature. What are the lessons of this paper that are not previously found in the literature?

This information has been added to the discussion as noted above.

The University of Newcastle
Callaghan NSW 2308 Australia
enquirycentre@newcastle.edu.au
CRICOS Provider Number: 00109J
T +61 2 4921 5000
www.newcastle.edu.au

- The effects of health assessments appears to be strongly coincident of a greater use of health services. One possible extension to further understand the effects of health assessments would be to perform a dose-response exercise to check if a great number of health assessments is associated with positive outcomes.

We agree, but since most women only had one assessment this is not possible. Instead, we have looked at when the assessments were delivered in terms of women's age, functional capacity, and mortality risk.

- Some parts of the propensity score matching section would benefit from some rewriting. For instance, the beginning of the section reads as "participants who had never had an assessment were assigned into blocks based on the date of their first health assessment".

This sentence has been corrected and simplified.

In addition, it would also be important to describe the list of covariates, squared terms, and interaction terms in a supplementary material to facilitate the reproduction of the paper's results.

Added – refer Supplementary Table 1

- One big concern of propensity matching (discussed by the authors in page 19) is that residual confounders may bias the results. It would be interesting to perform an "out of the sample" exercise as follows: select a characteristic or a set of characteristics that was not used in the propensity score model and check if there are statistically significant difference between participants who did and did not have health assessment.

See new Table 1. New Table 1 has been brought from Supplementary Materials into the manuscript to show balance after propensity matching.

Country of birth, alcohol consumption and needs help with daily living were not included in the propensity scores. These are balanced across the pairs after matching as seen in Table 1.

- The paper discusses that the matched sample is different from the original population. It would be interesting to know whether this difference might influence the key results of the paper.

A new Table 1 has been included showing the difference between the original cohort and the matched pairs. The differences are also considered in the discussion.

Note this table was previously included in supplementary materials

Minor Comments

The University of Newcastle
Callaghan NSW 2308 Australia
enquirycentre@newcastle.edu.au
CRICOS Provider Number: 00109J
T +61 2 4921 5000
www.newcastle.edu.au

- The manuscript would benefit from a proofreading review for typos, which can be also found in the abstract.

The manuscript has been proofread.

- I suggest improving the Figure 2 quality.

Please refer to the new 'Figure 2' file that will be uploaded separately with the revised manuscript.

- It seems the title describing Fig 3 has a comment in brackets that does not make sense. This comment might be misplaced, and it is likely that the comment refers to Fig 2.

Corrected.

- It is important to add that there are six survey rounds in the study.

Added.

- It was not clear in the "Outcome Variables Section" the years which healthcare utilization data are available

Dates are in the method section.

- It would be interesting to provide a brief intuition of the prognostic scores described on page 14. For instance, why does the score increase with age?

Prognostic scores are based on baseline variables which significantly predicted the hazards of death. These variables include age, area of residence, morbidity, GP visits and stiff or painful joints. Mortality risk increases with age. See Supplementary Table 2.

- On page 14, it seems that one of the ranges of the IQR has a typo.

These values are correct. In the low risk group, the median and upper IQR are the same, indicating that most women in the low-risk group lived the maximum amount of observed time.

- It seems that the results described in the last paragraph on page 16 are missing from Table 2.

The results for covariates "overweight" and "two or more conditions" are included in the text but not in the table. The table focusses on the primary analysis.

The University of Newcastle
Callaghan NSW 2308 Australia
enquirycentre@newcastle.edu.au
CRICOS Provider Number: 00109J
T +61 2 4921 5000
www.newcastle.edu.au

6. PLOS authors have the option to publish the peer review history of their article (what does this mean?). If published, this will include your full peer review and any attached files.

If you choose "no", your identity will remain anonymous but your review may still be made public.

Do you want your identity to be public for this peer review? For information about this choice, including consent withdrawal, please see our Privacy Policy.

Reviewer #1: No

Reviewer #2: No

[NOTE: If reviewer comments were submitted as an attachment file, they will be attached to this email and accessible via the submission site. Please log into your account, locate the manuscript record, and check for the action link "View Attachments". If this link does not appear, there are no attachment files.]

While revising your submission, please upload your figure files to the Preflight Analysis and Conversion Engine (PACE) digital diagnostic tool, https://pacev2.apexcovantage.com/. PACE helps ensure that figures meet PLOS requirements. To use PACE, you must first register as a user. Registration is free. Then, login and navigate to the UPLOAD tab, where you will find detailed instructions on how to use the tool. If you encounter any issues or have any questions when using PACE, please email PLOS at figures@plos.org. Please note that Supporting Information files do not need this step.

Please note that we have revised some of our figure files and are working on uploading these to the PACE digital diagnostic tool.

Sincerely,

Julie Byles

Professor Julie Byles BMed PhD FAAHMS https://www.newcastle.edu.au/profile/julie-byles

Global Innovation Chair in Responsive Transitions in Health and Ageing

Director, **Research Centre for Generational Health and Ageing**

School of Medicine and Public Health, Faculty of Health and Medicine

The University of Newcastle

Phone: +61 2 4042 0668 | Mob: +61 411 470 723 | Email: julie.byles@newcastle.edu.au
